# 

**Published:** 1914-12-15

**Authors:** 


### ANNOUNCEMENTS.

CHICAGO DENTAL SOCIETY.—Will hold its annual clinic and meeting for two days, January 29–30, 1915, at the Hotel La Salle in Chicago. Extensive preparations are on for a most interesting and profitable meeting.

NATIONAL ASSOCIATION OF DENTAL FACULTIES. Will hold its annual meeting in Ann Arbor, January 25–26, 1915. Headquarters at the Allenel Hotel. The meeting will open at 10 o'clock Monday, January 25th. Besides the regular business of the association several interesting papers of educational value will be read.

MONTANA BOARD OF EXAMINERS. Will hold a session for registration of dentists the second Monday in January, 1915.

AMERICAN INSTITUTE OF DENTAL TEACHERS. The annual meeting of the American Institute of Dental Teachers will be held at Ann Arbor, Michigan, January 26, 27 and 28th, 1915.

There will be a number of interesting papers, reports and discussions by prominent dental educators. All dental teachers are cordially invited to be present.

J. F. Biddle, *Secretary.*
517 Arch Street, N. S. Pittsburgh, Pa.

### NATIONAL DENTAL RELIEF FUND.

Office 63 Trumbull St.
New Haven, Conn.

To the Members of the National Dental Association:

The favorable response which you made last year in the introduction of a Christmas Seal, as a means to increase our Relief Fund, was most gratifying to your Committee. Realizing as we did, we were in a degree following the course of another organization, yet we felt assured, inasmuch as we were working for the same purpose, the relief of our suffering brothers, we should be, as we were, sustained by your liberal endorsement and contributions in excess of number of Seals sent you. We were handicapped last year, first by delay in having our design satisfactorily printed, stoppage by Post Master General, and much clerical expense, so we were unable to get the Seals in the hands of our members until about the first of December. However with all our disadvantage, we brought our fund up to $9,620. Profiting by experience, this year our expense will be nil, and the Seals can now be had at the Dental Supply houses, or this office. The necessity of a Relief Fund is made more manifest by repeated appeals to your committee by members who are suffering for the necessities of life, surely we should this year by our large increase in number, easily increase this Fund by purchase of Seals, and annual contributions which we are about to solicit, up to a sum, (not less than thirty thousand dollars), then from accrued interest, we could begin to respond to these calls from our unfortunate—and it's no exaggeration when we say suffering members. Brothers, a little from each will accomplish this much desired ideal, will you do it?

In sending your orders to this office, make all checks payable to National Dental Relief Fund, as your check becomes a receipt, saving expense in acknowledgment.

Fraternally yours,

L. G. Noel, W. T. Chambers,
James McManus, E. S. Gaylord, *Chairman.*

National Dental Relief Fund Committee.

